# miR‐516a‐3p inhibits breast cancer cell growth and EMT by blocking the Pygo2/Wnt signalling pathway

**DOI:** 10.1111/jcmm.14515

**Published:** 2019-07-05

**Authors:** Yanyan Chi, Feng Wang, Tengfei Zhang, Han Xu, Yana Zhang, Zhengzheng Shan, Shaoxuan Wu, Qingxia Fan, Yan Sun

**Affiliations:** ^1^ Department of Oncology The First Affiliated Hospital of Zhengzhou University Zhengzhou China; ^2^ Department of Breast Disease Surgery The First Affiliated Hospital of Zhengzhou University Zhengzhou China

**Keywords:** breast cancer, epithelial‐mesenchymal transition, miR‐516a‐3p, Pygo2, Wnt

## Abstract

miR‐516a‐3p has been reported to play a suppressive role in several types of human tumours. However, the expression level, biological function and fundamental mechanisms of miR‐516a‐3p in breast cancer remain unclear. In the present study, we found that miR‐516a‐3p expression was down‐regulated and Pygopus2 (Pygo2) expression was up‐regulated in human breast cancer tissues and cells. Through analysing the clinicopathological characteristics, we demonstrated that low miR‐516a‐3p expression or positive Pygo2 expression was a predictor of poor prognosis for patients with breast cancer. The results of a dual luciferase reporter assay and Western blot analysis indicated that Pygo2 was a target gene of miR‐516a‐3p. Moreover, overexpression of miR‐516a‐3p inhibited cell growth, migration and invasion as well as epithelial‐mesenchymal transition (EMT) of breast cancer cells, whereas reduced miR‐516a‐3p expression promoted breast cancer cell growth, migration, invasion and EMT. Furthermore, we showed that miR‐516a‐3p suppressed cell proliferation, metastasis and EMT of breast cancer cells by inhibiting Pygo2 expression. We confirmed that miR‐516a‐3p exerted an anti‐tumour effect by inhibiting the activation of the Wnt/β‐catenin pathway. Finally, xenograft tumour models were used to show that miR‐516a‐3p inhibited breast cancer cell growth and EMT via suppressing the Pygo2/Wnt signalling pathway. Taken together, these results show that miR‐516a‐3p inhibits breast cancer cell growth, metastasis and EMT by blocking the Pygo2/ Wnt/β‐catenin pathway.

## 1 INTRODUCTION

Breast cancer is one of the most common malignancies, ranking second among cancer‐related mortalities in women worldwide^1^ and is the most common malignancy in women in China,^2^ representing a serious threat to women's health. Although multiple strategies have been developed to treat breast cancer, such as surgery, radiotherapy and systemic chemotherapy, metastasis is still the main cause of death in breast cancer patients. Therefore, it is important to thoroughly investigate the molecular mechanisms of breast cancer metastasis and find potential biomarkers.

miRNAs are conserved noncoding RNAs that contain 18‐25 nucleotides and are associated with post‐transcriptional gene regulation.^3^ Emerging evidence shows that miRNAs participate in many biological processes, such as cell proliferation^4^ and apoptosis.^5^ A large number of studies have demonstrated that miRNAs can either promote the progression of cancer or act as tumour suppressors.^6^, ^7^ miRNAs also play crucial roles in tumour cell migration, invasion and epithelial‐mesenchymal transition (EMT).^8^, ^9^, ^10^, ^11^, ^12^, ^13^ The cancer‐related miRNA miR‐516a‐3p has been shown to be down‐regulated in gastric cancer^14^ and ovarian cancer,^15^ suggesting it has a role in suppressing human cancers. Yoshifumi Takei et al^14^ demonstrated that the expression of miR‐516a‐3p in 44As3 cells (highly metastatic gastric cancer cells) was lower than that in HSC‐44PE cells (parental cells isolated from patients), and sulfatase 1 was a direct target of the miR‐516a‐3p in gastric cancer. In vivo, miR‐516a‐3p markedly decreased metastases to the peritoneum. NMA White et al^15^ confirmed that miR‐516a‐3p can target kallikrein 10 (KLK10) and subsequently affect ovarian cancer cell proliferation. In this study, we investigated the role of miR‐516a‐3p in breast cancer for the first time.

Epithelial‐mesenchymal transition has been found to be closely related to tumour invasion and metastasis.^16^, ^17^ The molecular mechanisms of EMT are complex, and various molecules have been found to regulate EMT, including miRNAs.^18^, ^19^, ^20^, ^21^ Pygo2 has been shown to promote the proliferation of breast cancer cells.^22^ Zhang S et al^23^ reported that Pygo2 promoted cell invasion and metastasis through decreasing E‐cadherin expression in hepatic carcinoma. Additionally, Pygo2 is a novel functional protein, downstream of the Wnt signalling pathway.^24^ Pygo2 primarily binds to free β‐catenin to promote the progression of cancer by activating β‐catenin target genes, including cyclin D1 and c‐Myc.^22^, ^25^ Interestingly, the activation of the Wnt/β‐catenin signalling pathway has been reported to accelerate EMT in bladder cancer cells.^26^

In this study, we report for the first time the role of miR‐516a‐3p breast cancer. We demonstrated that miR‐516a‐3p inhibited breast cancer cell growth, metastasis and EMT by blocking the Pygo2/Wnt/β‐catenin signalling pathway both in vitro and in vivo.

## 2 MATERIALS AND METHODS

### 2.1 Clinical samples

Sixty paired breast cancer tissues and matched adjacent normal breast tissues were collected from the First Affiliated Hospital of Zhengzhou University between January and December 2013. The tissue samples were obtained from patients with pathologically verified breast cancer (all female; mean age 47 years; range 25‐69) who had undergone surgery with no patients having received chemotherapy or radiotherapy prior to surgery. All of the protocols used in this study were approved by the Medical Research Ethics Committee of the First Affiliated Hospital of Zhengzhou University. All of the patients signed informed consent. Samples were preserved in liquid nitrogen for quantitative real‐time polymerase chain reaction (qRT‐PCR) analysis or in formalin for immunohistochemical (IHC) staining. The clinical characteristics of the breast cancer patients were collected for analysis, including age, tumour size, lymph node metastasis, differentiation degree, molecular subtype and tumour‐node‐metastasis (TNM) tumour stage. The primary tumour (T), lymph node (N) and metastasis (M) classification of breast cancer was developed by the American Joint Commission of Cancer (AJCC).^27^ A follow‐up study was performed from 1 February 2013 to 1 June 2018. The follow‐up period was 8 to 64 months, and the median follow‐up time was 36 months. The follow‐up interviews were conducted by telephone. Five patients were lost, 13 patients died during the period and 42 patients survived until the end of the follow‐up period.

### 2.2 Cell culture and transfection

Normal breast cell line (HBL‐100), breast cancer cell lines (MDA‐MB‐231 and MCF‐7) and HEK293T cells were purchased from the Chinese Academy of Sciences Type Culture Collection. All cell lines were cultured in DMEM (HyClone) containing 10% foetal bovine serum (FBS), and 1% penicillin‐streptomycin at 37°C with 5% CO_2_.

miR‐516a‐3p mimic, miR‐516a‐3p inhibitor, relevant negative controls, Pygo2 siRNA, pcDNA3.1‐Pygo2 plasmid and pcDNA3.1‐vector plasmid were provided by GenePharma. Lipofectamine 3000 reagent (Invitrogen) and Opti‐MEM (Gibco) were used to transfect the cells following the manufacturer's instructions. MDA‐MB‐231 and MCF‐7 cells were seeded into 6‐well plates and cultured for 24 hours at 37°C with 5% CO_2_. Lipofectamine 3000 reagent was diluted with Opti‐MEM. miR‐516a‐3p mimic, miR‐516a‐3p inhibitor, pcDNA3.1‐Pygo2 plasmid or Pygo2 siRNA were dissolved with Opti‐MEM, added into diluted Lipofectamine 3000 and incubated for 5 minutes at room temperature. Subsequently, the cells were added with the mixtures and serum‐free medium.

### 2.3 RNA extraction and qRT‐PCR

RNA was extracted from tissue samples or cells using TRIzol Reagent (TaKaRa) following the manufacturer's instructions. A Mir‐X miRNA First‐Strand Synthesis Kit (TaKaRa) and PrimeScript RT Master Mix (TaKaRa) were used for reverse transcription. qRT‐PCR was performed using SYBR Premix Ex Taq (Roche) to detect the relative expression of miR‐516a‐3p and Pygo2. The specific primers used were synthesized by Sangon (Table 1). The 2^(−ΔΔCT)^ method was used to calculate the level of miR‐516a‐3p and Pygo2 expression. U6 and GAPDH were regarded as controls for miR‐516a‐3p and Pygo2, respectively.

**Table 1:** The primer sequences used in qRT‐PCR

Gene	Sequence
miR‐516a‐3p‐F	5′‐GCTGCTTCCTTTCAGAGGGT‐3′
GAPDH‐F	5′‐CTCCTCCACCTTTGACGCTG‐3′
GAPDH‐R	5′‐CATACCAGGAAATGAGCTTGACAA‐3′
Pygo2‐F	5′‐GTTTGGGCTGTCCTGAAAGTCTG‐3′
Pygo2‐R	5′‐ATAAGGGCGCCGAAAGTTGA‐3′

Abbreviations: F, forward primer; R, reverse primer.

### 2.4 Immunohistochemical staining and scoring

Tissues were paraffin‐embedded and sliced into 4‐μm thick sections for Pygo2 (1:50, Abcam), E‐cadherin (1:400, CST, USA) or vimentin (1:400, Abcam) staining. IHC staining was performed as previously described.^26^ IHE staining was scored according to the proportion of positively stained cells: 0, 0%; 1, 1%‐25%; 2, 26%‐50%; 3, 51%‐75%; and 4, >75%.

### 2.5 Western blotting

Proteins were extracted from breast cancer tissues or cells using RIPA lysis buffer supplemented with protease inhibitor. Next, total proteins were separated using gel electrophoresis and transferred to polyvinylidene fluoride (PVDF) membranes (Beyotime) via electroblotting. The PVDF membranes were blocked with 5% skim milk, after which they were incubated with primary antibodies rabbit anti‐Pygo2 (1:1000, Abcam), rabbit anti‐E‐cadherin (1:1000, CST), rabbit anti‐vimentin (1:2000, Abcam), rabbit anti‐β‐catenin (1:1000, Abcam), rabbit anti‐c‐Myc (1:1000, Abcam), rabbit anti‐cyclin D1 (1:1000, Abcam) and rabbit anti‐GAPDH (1:4000, Santa Cruz) overnight at 4°C. Subsequently, the membranes were incubated with the appropriate secondary antibodies, including goat antimouse IgG (1:10 000, Affinity) and goat anti‐rabbit IgG (1:10 000, Affinity), for 1 hour. ECL detection reagent (Santa Cruz) was added on the membranes to detect signals. GAPDH used as a loading control. The greyscale values of protein bands were analysed using ImageJ.

### 2.6 Dual luciferase reporter assay

The potential target genes of miR‐516a‐3p were predicted with bioinformatic algorithms from the publicly available databases TargetScan (http://www.targetscan.org) and http://mirtarbase.mbc.nctu.edu.tw/php/index.php. The wild‐type 3’UTR of Pygo2 mRNA sequence containing the predicted target sites of miR‐516a‐3p was synthesized and is shown in Figure 2A. The reporter vectors containing Pygo2 wild‐type (pmirGLO‐Pygo2‐wt) and Pygo2 mutant miR‐516a‐3p‐binding sequence, (pmirGLO‐Pygo2‐mut) or the negative control sequence (pmirGLO‐NC) were provided by Gene Pharm. A dual luciferase reporter assay was performed as previously described.^28^ pmirGLO‐Pygo2‐wt vector, pmirGLO‐Pygo2‐mut vector or pmirGLO‐NC vector was cotransfected into HEK293T cells with miR‐516a‐3p mimic or miR‐516a‐3p negative control. After 24 hours, activities of firefly luciferase and Renilla luciferase were detected according to the manufacturer's instruction for the Dual Luciferase Reporter Assay System (Promega).

### 2.7 Proliferation assay

Cells were passed in 96‐well plates at a density of 2000 cells/well after transfection. Cell viability was determined using the Cell Counting Kit‐8 (CCK‐8) assay. The principle of the CCK‐8 assay is the same as that of the 3‐(4, 5‐dimethylthiazol‐2‐yl)‐2, 5‐diphenyltetrazolium bromide (MTT) assay.^29^ However, because the formazan produced using CCK‐8 method is water‐soluble, steps involving removal of culture medium and the addition of organic solvent do not need to be performed, reducing error. The cells were added with CCK‐8 solution and incubated for 1 hour at 37°C. The proliferation capacity of cells was shown by the ratio of absorbance, measured at 450 nm of the test group to that of control cells.

#### 2.7.1 Cell apoptosis assay

Cell apoptosis was measured by flow cytometry using an Annexin V‐FITC/PI apoptosis detection kit (BD) on the basis of the manufacturer's protocol. Transfected cells were harvested and stained with Annexin V and PI. Samples were analysed using a flow cytometer.

### 2.8 Wound healing assay

Wound healing assay was used to detect the migration of MDA‐MB‐231 and MCF‐7 cells. Transfected cells were cultured in 6‐well plates until the conflux reached 80%. The cell layer in each well was wounded by scratching the cells with aseptic 10 μL plastic pipette tips. Images of the scratches were captured at 0 and 48 hours.

### 2.9 Transwell migration/invasion assays

Cell migration and invasion were measured with a 24‐well transwell plate (Corning). The upper chamber was added with transfected cells suspended in serum‐free medium (1 × 10^5^ cells/chamber for MCF‐7 cells and 1.5 × 10^4^ cells/chamber for MDA‐MB‐231 cells) and the lower chamber was added with 600 µL culture medium containing 20% FBS. Cells were incubated at 37°C for 10 hours. Then cells were fixed with 4% paraformaldehyde for 20 minutes and stained with 0.1% crystal violet for 30 minutes. The unmigrated cells on the upper surface of the film were removed by cotton swabs, and the migrated cells on lower surface of the film were observed and counted under a microscope. For the transwell invasion assay, Matrigel (BD Biosciences) was diluted and placed in the upper chamber.

### 2.10 Mouse xenograft model

Animal experiment was approved by the Institutional Ethics Review Board of Zhengzhou University. The right‐side fat pads of eighteen 5‐week‐old female BALB/c nude mice were injected with MDA‐MB‐231 cells (1 × 10^6^/100 μl/site). After 6 days, the mice were randomly divided into three groups, including the blank control group, the negative control group and the miR‐516a‐3p group. The tumours of mice were injected with either PBS, an angomir control or miR‐516a‐3p angomir every other day. Tumour size was recorded every three days and the primary tumours were removed 4 weeks after cells were injected. The tumours were stored in liquid nitrogen for Western blotting or in formalin for terminal deoxynucleotidyl transferase dUTP nick‐end labelling analysis (TUNEL) and IHC staining.

### 2.11 TUNEL

Apoptotic cells in the breast cancer tumour sections from mice were detected via a TUNEL assay. Sections were dewaxed in xylene for 5‐10 minutes and then were dewaxed in fresh xylene for 5‐10 minutes. Sections were placed in absolute ethanol for 5 minutes, 95% ethanol for 2 minutes, 85% ethanol for 2 minutes, 75% ethanol for 2 minutes and distilled water for 2 minutes. Next, the tissues were incubated with 2% DNase‐free proteinase K at 37°C for 20 minutes, washed them with PBS three times and then were blocked with 5% serum at 37°C for 20 minutes. The sections were treated following the manufacturer's instructions for the In Situ Cell Death Detection Kit (Roche Applied Science). Images were acquired under a fluorescence microscope (Germany Lecia photomicrography system) and Image‐Pro Plus (Media Cybernetics) was used to evaluate positive staining in the sections.

### 2.12 Statistical analysis

SPSS (version 21.0; SPSS, Inc) was used to analyse the data. The miRNA expression and Pygo2 expression in paired tumour and adjacent normal tissues were determined using the paired two‐sample Student's *t* test. The data among the groups were detected by the Student's *t* test or a one‐way analysis of variance (ANOVA) and shown as the means ± standard deviation. Correlations between clinicopathological parameters and miR‐516a‐3p or Pygo2 expression were analysed with chi‐squared test. Survival analysis was determined using Kaplan‐Meier plots and log‐rank tests. Differences with *P* < 0.05 were regarded as significance.

## 3 RESULTS

### 3.1 miR‐516a‐3p expression is down‐regulated and inversely correlated with Pygo2 expression in human breast cancer tissue and cell lines

**Figure 1:**
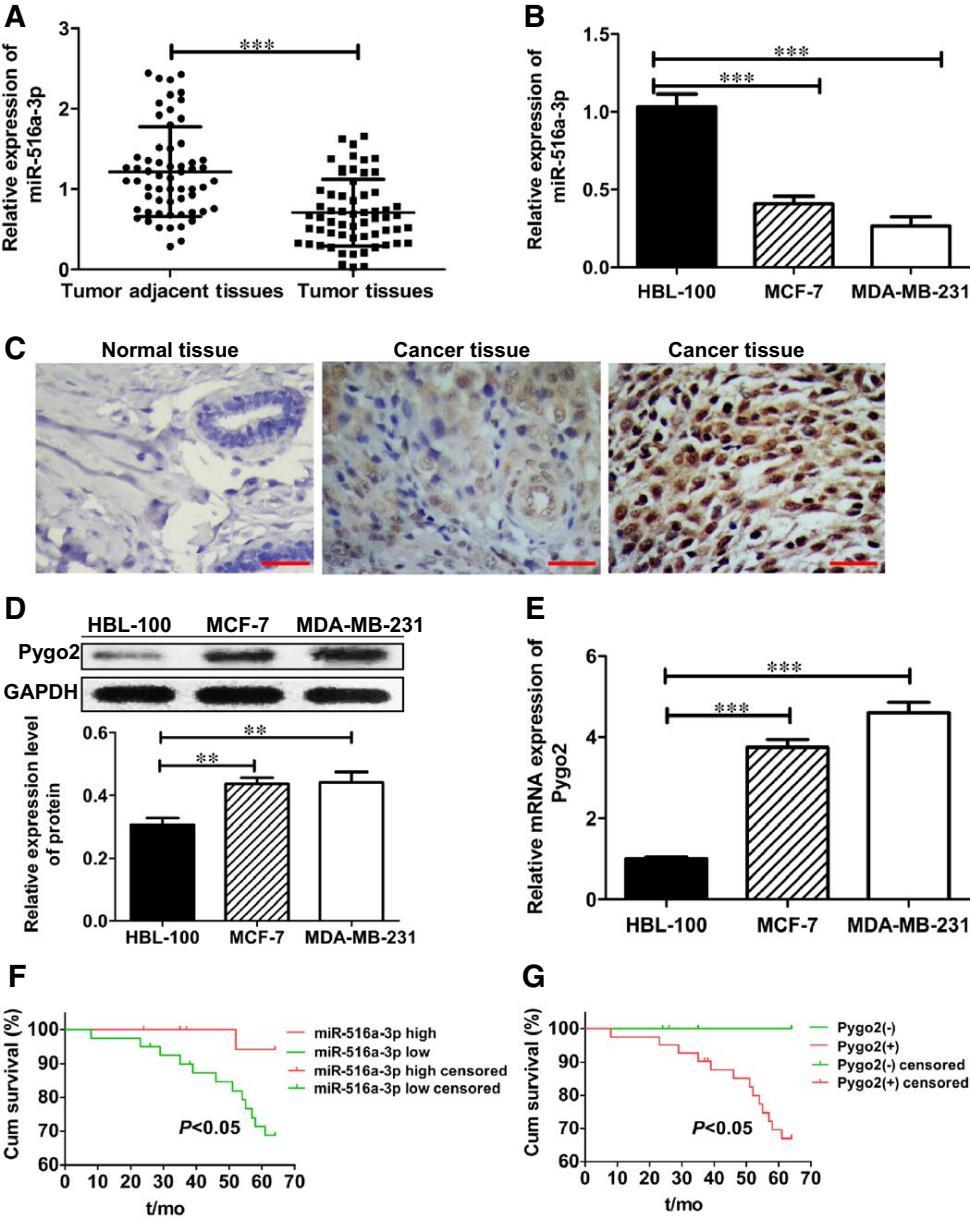
miR‐516a‐3p is down‐regulated and Pygo2 is up‐regulated in breast cancer tissues and cells. A, miR‐516a‐3p expression was compared between breast cancer and paired adjacent normal breast tissues (n = 60). B, miR‐516a‐3p expression in human breast cancer cell lines (MCF‐7 and MDA‐MB‐231) and in normal human breast cell line (HBL‐100). C, Negative expression of Pygo2 protein in adjacent normal breast tissues (×400). Weak positive expression of Pygo2 protein in breast cancer tissues (×400). Strong positive expression of Pygo2 protein in breast cancer tissues (×400), bar = 50 μm, n = 60. (D‐E) The expression of Pygo2 protein and mRNA in various human breast cancer cell lines (MCF‐7 and MDA‐MB‐231) and in normal human breast cell line (HBL‐100). F, OS was compared between breast cancer patients with a high miR‐516a‐3p expression level and those with a low miR‐516a‐3p expression level. G, OS was compared between breast cancer patients with positive expression of Pygo2 protein and those with negative expression of Pygo2 protein. Data are shown as mean ± SD (**, *P* < 0.01; ***, *P* < 0.001)

To assess the expression level of miR‐516a‐3p and Pygo2 in breast cancer, we detected their expression in 60 paired breast cancer tissue and matched normal breast tissue samples. qRT‐PCR results showed that miR‐516a‐3p expression was significantly down‐regulated in most of the breast cancer tissue samples compared with that in the matched controls (Figure 1A). IHC staining results showed that Pygo2 protein expression was up‐regulated in 68% (41/60) of the breast cancer tissue samples (Table 2, Figure 1B). In the cell lines, we found miR‐516a‐3p expression was lower in breast cancer cells MCF‐7 and MDA‐MB‐231 than that in the normal breast cell line HBL‐100 (Figure 1C), whereas Pygo2 protein and mRNA expression were higher in breast cancer cells MCF‐7 and MDA‐MB‐231 than that in the normal breast cells HBL‐100 (Figure 1D‐E). These data show that the miR‐516a‐3p expression is down‐regulated and Pygo2 expression is up‐regulated in breast cancer.

**Table 2:** Correlation between clinicopathological characteristics and expression of miR‐516a‐3p and Pygo2 in patients with breast cancer

Clinicopathological characteristics	n	miR‐516a‐3pexpression		*X^2^*	*P*	Pygo2 expression		*X^2^*	*P*
		High	Low			Positive	Negative		
Age									
≤50	27	10	17	**0.303**	**0.582**	17	10	**0.654**	**0.419**
>50	33	10	23			24	9		
Tumour size									
T ≤ 2 cm	36	17	19	**7.813**	**0.005**	21	15	**4.159**	**0.041**
T > 2 cm	24	3	21			20	4		
Lymph node status									
Negative	42	19	23	**8.929**	**0.003**	25	17	**5.021**	**0.025**
Positive	18	1	17			16	2		
Differentiation									
Well	28	10	18	**1.107**	**0.575**	19	9	**1.018**	**0.601**
Moderate	15	6	9			10	5		
Poor	17	4	13			12	5		
TNM tumour stage									
I + II	43	19	24	**8.044**	**0.005**	26	17	**4.342**	**0.037**
III + IV	17	1	16			15	2		
Molecular subtype									
Luminal A	36	13	23	**0.293**	**0.725** a	24	12	**0.925**	**0.464** b
Luminal B	5	1	4			3	2		
HER‐2(+)	7	3	5			5	3		
Basal‐like	12	3	8			9	2		

a miR‐516a‐3p expression in luminal A compared with basal‐like.

b Pygo2 expression in luminal A compared with basal‐like.

The bold indicates the significance value.

### 3.2 Low miR‐516a‐3p expression or positive Pygo2 expression is a predictor of poor prognosis for patients with breast cancer

As shown in Table 2, the low level of miR‐516a‐3p expression in breast cancer tissues compared to the matched normal breast tissues was markedly related to lymph node metastasis (*P* = 0.003), increased tumour size (*P* = 0.005) and worse TNM stage (*P* = 0.005, Table 2). Meanwhile, positive expression of Pygo2 was also related to lymph node metastasis (*P* = 0.025), increased tumour size (*P* = 0.041) and worse TNM stage (*P* = 0.037). In both luminal A and basal‐like of breast cancer, the expression of miR‐516a‐3p was lower and positive rate of Pygo2 expression was higher than that in adjacent normal breast tissues. However, there was no significant correlation between the expression of miR‐516a‐3p/Pygo2 and molecular subtypes (*P* > 0.05, Table 2). Furthermore, compared with patients with high miR‐516a‐3p levels, patients with low miR‐516a‐3p level had shorter overall survival (OS; *P* < 0.05, Figure 1F). Additionally, the OS for patients with positive Pygo2 expression was markedly worse than for those patients with negative Pygo2 expression (*P* < 0.05, Figure 1G). These results indicate that lower miR‐516a‐3p expression or positive Pygo2 expression is related to poor clinical features in breast cancer patients. These results show that both miR‐516a‐3p and Pygo2 have the potential to be prognostic biomarkers for breast cancer.

### 3.3 Pygo2 is a target gene of miR‐516a‐3p

**Figure 2:**
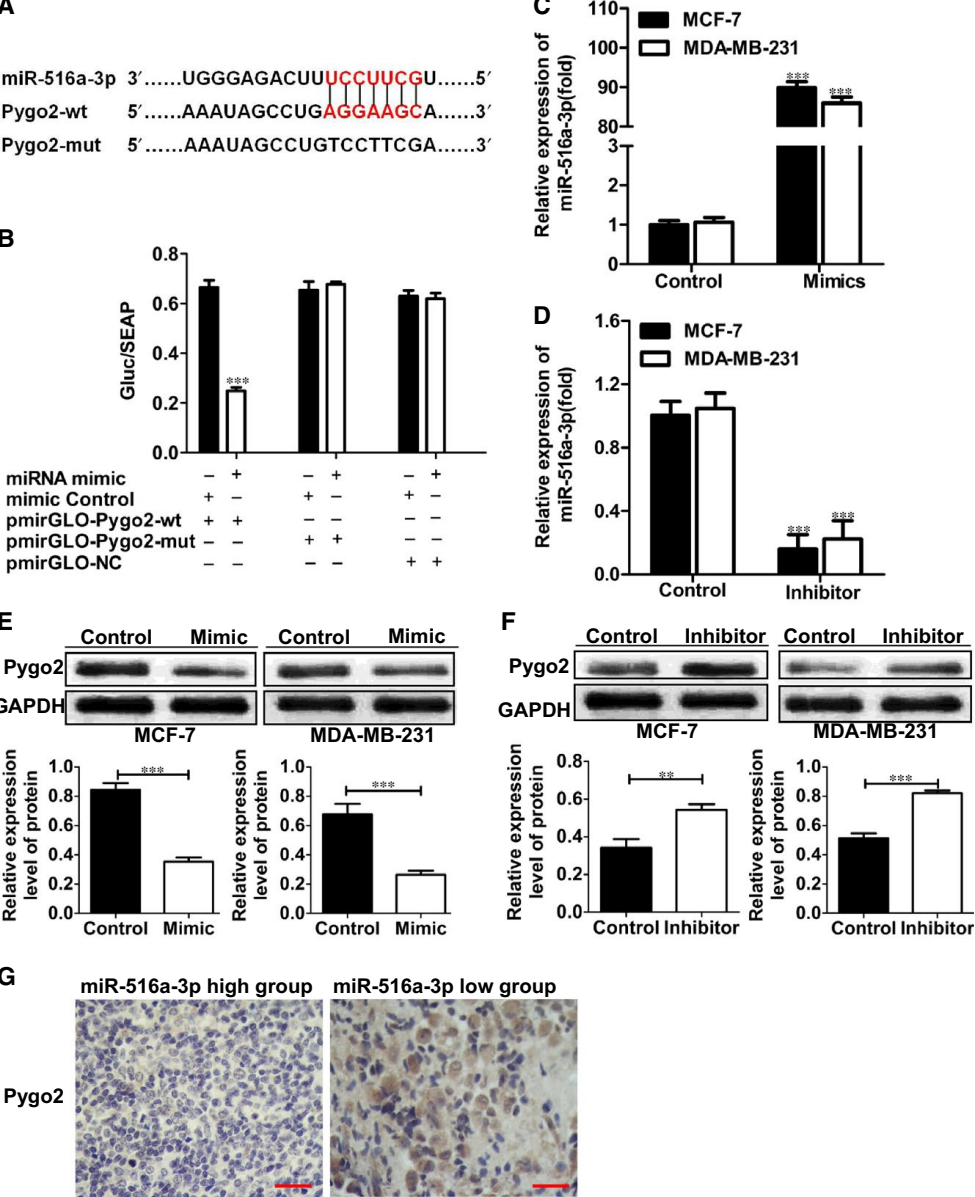
Pygo2 is a target gene of miR‐516a‐3p. A, miR‐516a‐3p and its putative binding sequence in the 3′‐UTR of Pygo2 mRNA. Mutant miR‐516a‐3p binding sites were generated in the complementary site for the seed region of miR‐516a‐3p (WT, wild type; Mut, mutant type). B, miR‐516a‐3p effects on luciferase activity in cells that carried the wild type and mutant type 3’‐UTR of Pygo2 mRNA. (C‐D) miR‐516a‐3p expression was changed after transfection of MCF‐7 and MDA‐MB‐231 cells with miR‐516a‐3p mimic or inhibitor. E, Western blot analysis of the expression of Pygo2 protein in MCF‐7 and MDA‐MB‐231 breast cancer cell lines transfected with miR‐516a‐3p NC or miR‐516a‐3p mimic. F, Western blot analysis of the expression of Pygo2 protein in MCF‐7 and MDA‐MB‐231 breast cancer cell lines transfected with miR‐516a‐3p NC or miR‐516a‐3p inhibitor. G, Immunohistochemistry of Pygo2 was shown and compared between tissues with high miR‐516a‐3p level and those with low miR‐516a‐3p level (×400), bar = 50 μm. Data are shown as mean ± SD (**, *P* < 0.01; ***, *P* < 0.001)

Bioinformatics analysis predicted Pygo2 as a potential target gene of miR‐516a‐3p (Figure 2A). To confirm this result, a dual luciferase reporter assay was carried out. HEK293T cells were transfected with pmirGLO‐Pygo2‐wt reporter vector, pmirGLO‐Pygo2‐mut reporter vector or pmirGLO‐NC along with miR‐516a‐3p mimic or miR‐516a‐3p negative control. Results demonstrated that cotransfection of pmirGLO‐Pygo2‐wt and miR‐219‐5p mimic significantly decreased luciferase activity compared with the control group, whereas cotransfection of pmirGLO‐Pygo2‐mut/pmirGLO‐NC and miR‐219‐5p mimic had no effect on luciferase activity (Figure 2B).

In order to further explore the relationship between miR‐516a‐3p and Pygo2, MCF‐7 and MDA‐MB‐231 cells were transfected with miR‐516a‐3p mimic or miR‐516a‐3p inhibitor to up‐regulate or down‐regulate miR‐516a‐3p expression, respectively. qRT‐PCR analysis showed that miR‐516a‐3p mimic and miR‐516a‐3p inhibitor successfully increased and decreased the expression of miR‐516a‐3p in breast cancer cells, respectively (Figure 2C‐D). We tested the Pygo2 protein expression in MCF‐7 and MDA‐MB‐231 cells transfected with miR‐516a‐3p mimic and inhibitor using Western blot. The results demonstrated that cells treated with miR‐516a‐3p mimic showed decreased Pygo2 protein expression compared with control group (Figure 2E), and cells treated with miR‐516a‐3p inhibitor exhibited increased Pygo2 protein expression compared with control group (Figure 2F). IHC staining results showed that the level of Pygo2 protein in breast cancer tissues exhibiting high miR‐516a‐3p expression was lower than that in breast cancer tissues with low miR‐516a‐3p expression. (Figure 2G) These data indicate that Pygo2 is a target gene of miR‐516a‐3 in breast cancer.

### 3.4 miR‐516a‐3p inhibits breast cancer cell growth in vitro

**Figure 3:**
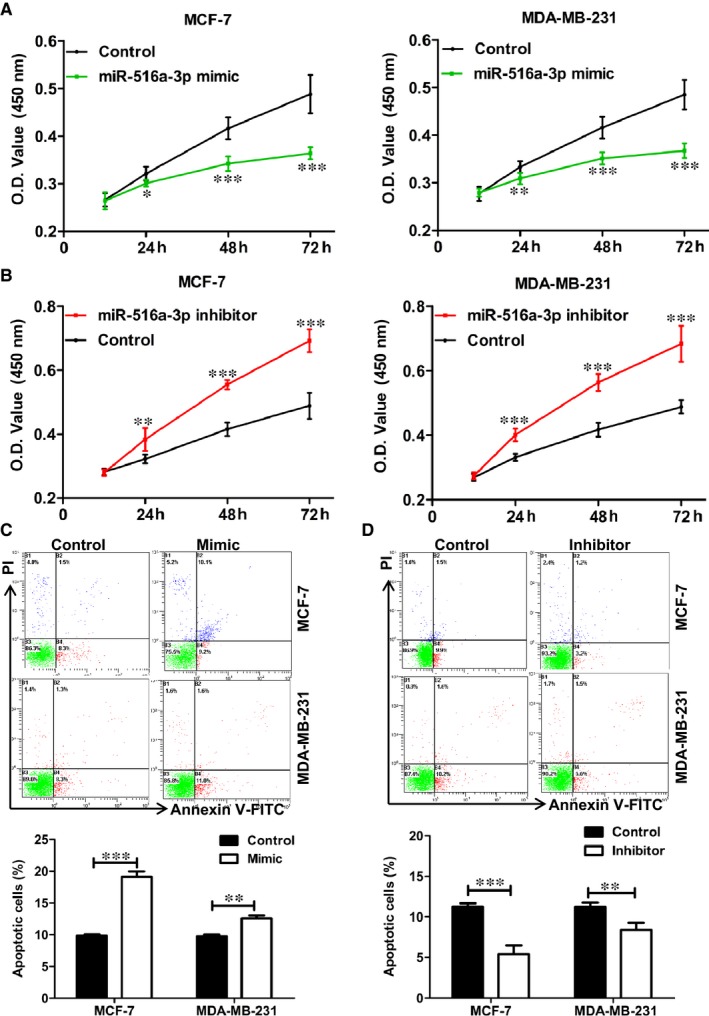
miR‐516a‐3p inhibits breast cancer cell growth in vitro. A, The proliferation ability of MCF‐7 and MDA‐MB‐231 cells after treatment with miR‐516a‐3p mimic was examined with a CCK‐8 kit. B, The proliferation ability of MCF‐7 and MDA‐MB‐231 cells after treatment with miR‐516a‐3p inhibitor was examined with a CCK‐8 assay. C, The cell apoptosis was analyzed in MCF‐7 and MDA‐MB‐231 cells transfected with miR‐516a‐3p NC or miR‐516a‐3p mimic. D, The cell apoptosis was analyzed in MCF‐7 and MDA‐MB‐231 cells transfected with miR‐516a‐3p NC or miR‐516a‐3p inhibitor. Data are shown as mean ± SD (*, *P* < 0.05; **, *P* < 0.01; ***, *P* < 0.001)

To explore the effect of miR‐516a‐3p on breast cancer growth in vitro, we performed the CCK‐8 assay and flow cytometry to detect cell proliferation and cell apoptosis. CCK‐8 assay results showed that the proliferation of MCF‐7 and MDA‐MB‐231 cells was inhibited after transfecting with miR‐516a‐3p mimic compared with that of the control group (Figure 3A), while decreased miR‐516a‐3p expression showed increased cell proliferation in vitro (Figure 3B). Moreover, overexpression of miR‐516a‐3p significantly induced cell apoptosis in both MCF‐7 and MDA‐MB‐231 cells (Figure 3C), whereas inhibition of miR‐516a‐3p attenuated these effects (Figure 3D).

### 3.5 miR‐516a‐3p inhibits migration, invasion and EMT of breast cancer cells

**Figure 4:**
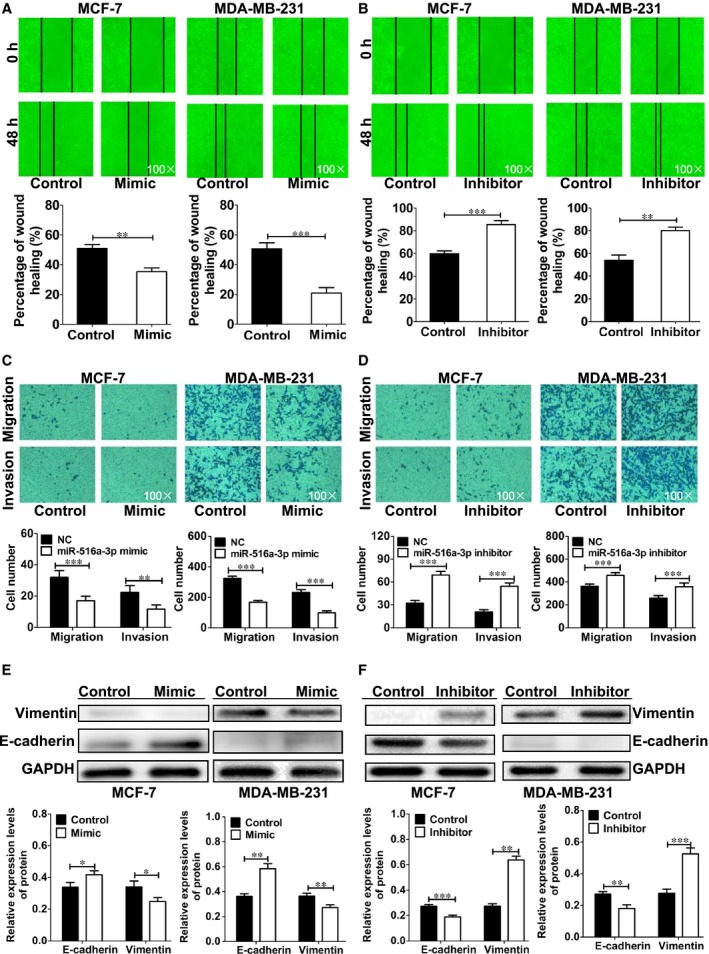
miR‐516a‐3p inhibits migration, invasion and EMT of breast cancer cells. A, Wound healing assay results showed that miR‐516a‐3p overexpression decreased MCF‐7 and MDA‐MB‐231 cell migration (×100). B, Wound healing assay results showed that miR‐516a‐3p inhibition increased MCF‐7 and MDA‐MB‐231 cell migration (×100). C, Transwell assay results showed that miR‐516a‐3p overexpression decreased MCF‐7 and MDA‐MB‐231 cell migration and invasion (×100). D, Transwell assay results showed that miR‐516a‐3p inhibition increased MCF‐7 and MDA‐MB‐231 cell migration and invasion (×100). E, Western blot analysis of E‐cadherin and vimentin protein expression in MCF‐7 and MDA‐MB‐231 breast cancer cell lines transfected with miR‐516a‐3p NC or miR‐516a‐3p mimic. F, Western blot analysis of E‐cadherin and vimentin protein expression in MCF‐7 and MDA‐MB‐231 breast cancer cell lines transfected with miR‐516a‐3p NC or miR‐516a‐3p inhibitor. Data are shown as mean ± SD (**, *P* < 0.01; ***, *P* < 0.001)

To confirm the suppressive metastasis role of miR‐516a‐3p, we evaluated cell migration and invasion using wound healing and transwell assays. The results showed that migration and invasion were significantly inhibited after cells were transfected with miR‐516a‐3p mimic compared with that of the control group (Figure 4A, 4). On the other hand, migration and invasion were significantly increased after breast cancer cells were treated with miR‐516a‐3p inhibitor (Figure 4B, 4). These data show that miR‐516a‐3p inhibits breast cancer cell migration and invasion.

Epithelial‐mesenchymal transition is a crucial process for tumour invasion and metastasis. To investigate the effect of miR‐516a‐3p on EMT, MCF‐7 and MDA‐MB‐231 cells were transfected with miR‐516a‐3p mimic, miR‐516a‐3p inhibitor or negative control. Western blot results demonstrated that miR‐516a‐3p overexpression induced increased E‐cadherin protein expression and decreased vimentin protein expression (Figure 4E). On the other hand, we found that miR‐516a‐3p reduction decreased E‐cadherin protein expression and increased vimentin protein expression (Figure 4F). These results indicate that miR‐516a‐3p inhibits EMT in breast cancer, and further confirm that it is a suppressor of metastasis in breast cancer cells.

### 3.6 miR‐516a‐3p inhibits cell proliferation, migration, invasion and EMT by blocking Pygo2 expression in breast cancer cells

**Figure 5:**
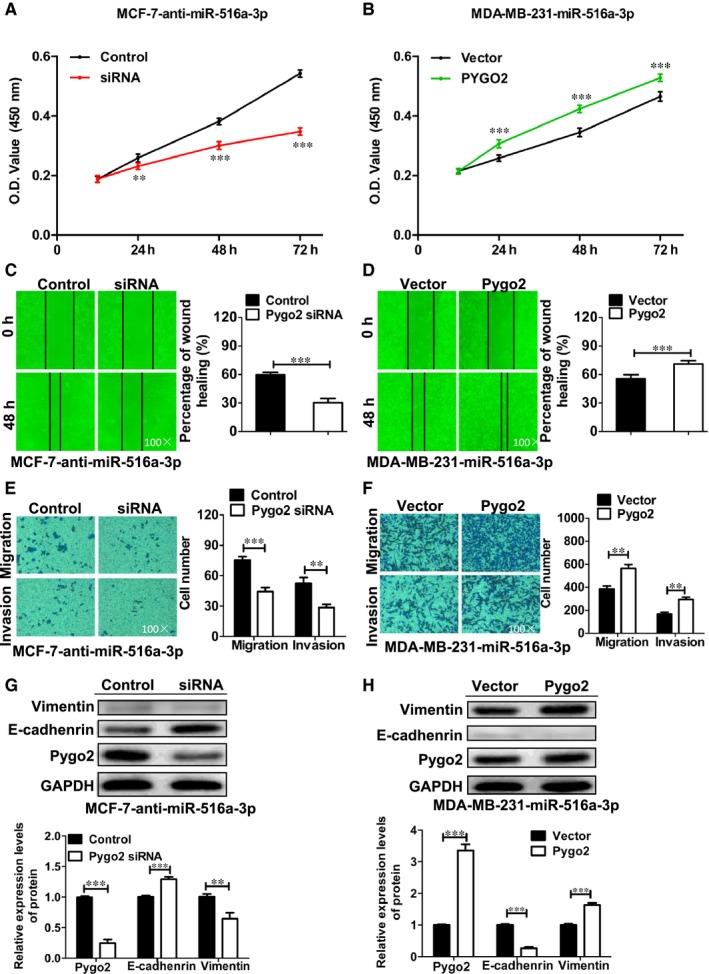
miR‐516a‐3p inhibits cell proliferation, migration, invasion and EMT by blocking Pygo2 in breast cancer cells. A, CCK‐8 assay for MCF‐7 cells expressing miR‐516a‐3p inhibitor transfected with Pygo2 siRNA or NC siRNA. B, CCK‐8 assay for MDA‐MB‐231 cells overexpressing miR‐516a‐3p transfected with pcDNA3.1‐Pygo2 plasmid or control vector. C, Wound healing assay for MCF‐7 cells expressing miR‐516a‐3p inhibitor transfected with Pygo2 siRNA or NC siRNA (×100). D, Wound healing assay for MDA‐MB‐231 cells overexpressing miR‐516a‐3p transfected with pcDNA3.1‐Pygo2 plasmid or control vector (×100). E, Transwell assay for MCF‐7 cells expressing miR‐516a‐3p inhibitor transfected with Pygo2 siRNA or NC siRNA (×100). F, Transwell assay for MDA‐MB‐231 cells overexpressing miR‐516a‐3p transfected with pcDNA3.1‐Pygo2 plasmid or control vector (×100). G, Western blot analysis of the Pygo2, E‐cadherin and vimentin protein expression in MCF‐7 cells expressing miR‐516a‐3p inhibitor transfected with Pygo2 siRNA or NC siRNA. H, Western blot analysis of the Pygo2, E‐cadherin and vimentin protein expression in MDA‐MB‐231 cells expressing miR‐516a‐3p mimic transfected with pcDNA3.1‐Pygo2 plasmid or control vector. Data are shown as mean ± SD (*, *P* < 0.05; **, *P* < 0.01; ***, *P* < 0.001)

We further investigated the mechanism of miR‐516a‐3p inhibiting cell proliferation and EMT of breast cancer cells. The silencing Pygo2 expression repealed the increased cell proliferation, migration and invasion of MCF‐7 cells induced by miR‐516a‐3p downregulation (Figure 5A, 5, 5). Furthermore, Pygo2 siRNA significantly decreased Pygo2 protein expression in MCF‐7 cells transfected with miR‐516a‐3p inhibitor. The silencing of Pygo2 expression reversed the EMT induced by miR‐516a‐3p inhibition in MCF‐7 cells. In addition, Western blotting analysis showed that Pygo2 siRNA increased E‐cadherin protein expression and decreased vimentin protein expression (Figure 5G). On the other hand, overexpression of Pygo2 nullified the inhibitory role of miR‐516a‐3p mimic in the cell proliferation, migration and invasion of MDA‐MB‐231 cells (Figure 5B, 5, 5). Pygo2 overexpression increased Pygo2 protein expression and reversed the mesenchymal‐epithelial transition of MDA‐MB‐231 cells induced by miR‐516a‐3p mimic (Figure 5H). These results demonstrate that miR‐516a‐3p inhibits cell proliferation and EMT through blocking Pygo2 expression in breast cancer.

### 3.7 miR‐516a‐3p inhibits activity of the Wnt/β‐ catenin pathway of breast cancer cells

**Figure 6:**
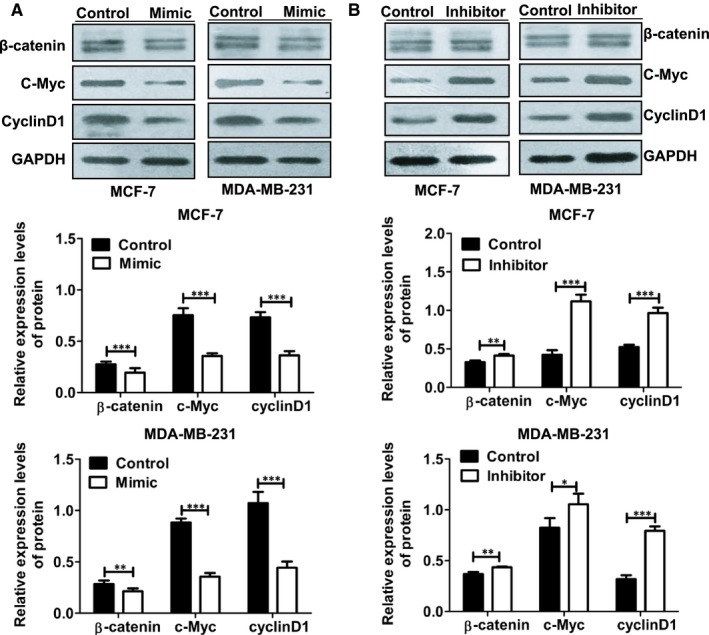
miR‐516a‐3p reduces indicated protein expression of the Wnt pathway in human breast cancer cells. A, Western blot analysis of the β‐catenin, c‐Myc and cyclinD1 protein expression in MCF‐7 and MDAMB‐231 breast cancer cell lines transfected with miR‐516a‐3p NC or miR‐516a‐3p mimic. B, Western blot showing the β‐catenin, c‐Myc, and cyclinD1 protein expression in MCF‐7 and MDA‐MB‐231 breast cancer cell lines transfected with miR‐516a‐3p NC or miR‐516a‐3p inhibitor. Data are shown as mean ± SD (*, *P* < 0.05; **, *P* < 0.01; ***, *P* < 0.001)

Pygo2 is a novel functional protein downstream of the Wnt signalling pathway which is also related to EMT. To further study whether miR‐516a‐3p has an effect on the Wnt pathway, we detected key molecules in the Wnt signalling pathway. miR‐516a‐3p overexpression markedly reduced β‐catenin, c‐Myc and cyclin D1 expression (Figure 6A), while miR‐516a‐3p depletion enhanced β‐catenin, c‐Myc and cyclin D1 expression (Figure 6B). These data indicate that miR‐516a‐3p inhibits activity of the Wnt/β‐catenin pathway.

### 3.8 miR‐516a‐3p inhibits breast cancer cells growth and EMT by blocking the Pygo2/Wnt signalling pathway in vivo

**Figure 7:**
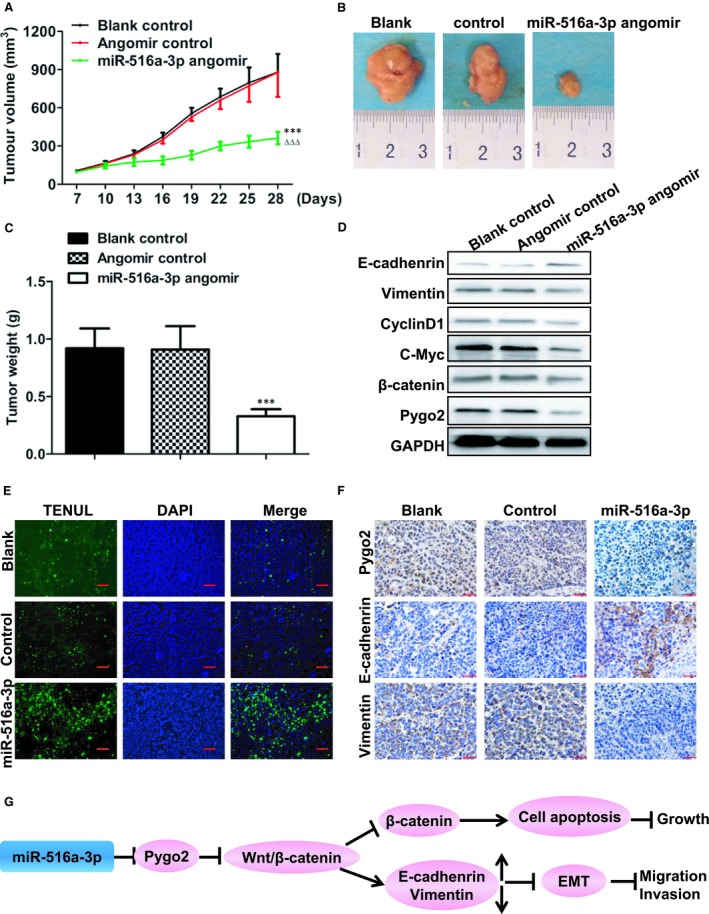
miR‐516a‐3p inhibits breast cancer cell growth and EMT by blocking Pygo2/Wnt signalling pathway in vivo. A, The average of tumour volumes was plotted, n = 6. B, Xenograft tumours were resected from mice after 4 weeks post‐cells injection, n = 6. C, Tumour weights of different groups at day 30, n = 6. D, Western blot showing the E‐cadherin, vimentin, Pygo2, β‐catenin, c‐Myc, and CyclinD1 protein expression in tumour tissues. E, Tunel results showed the rate of cell apoptosis in breast cancer tumour tissues treated with PBS, angomir control or miR‐516a‐3p angomir (×400), bar = 50 μm, n = 6. F, Immunohistochemistry results showed the expression of E‐cadherin, Pygo2 and vimentin protein in breast cancer tumour tissues treated with PBS, angomir control, or miR‐516a‐3p angomir (×400), bar = 50 μm, n = 6. G, A schematic sketch of miR‐516a‐3p functions in breast cancer. Data are shown as mean ± SD (***, *P* < 0.001, miR‐516a‐3p angomir group compared with angomir control group; ΔΔΔ, *P* < 0.001, miR‐516a‐3p angomir group compared with blank control group)

To explore the effect of miR‐516a‐3p on breast cancer cell growth in vivo, MDA‐MB‐231 cells were inoculated into the right‐side fat pads of female BALB/c nude mice. After 6 days, the tumours of mice were separately injected into PBS, angomir control and miR‐516a‐3p angomir every other day. After 3 weeks, the tumours injected with the miR‐516a‐3p angomir showed potent tumour growth inhibition (Figure 7A‐B) with the weights of the tumours in miR‐516a‐3p angomir group being lower than those in angomir control group and blank group (Figure 7C).

Western blot analysis showed that E‐cadherin expression was significantly increased by miR‐516a‐3p angomir compared with angomir control and PBS. Vimentin, Pygo2, β‐catenin, c‐Myc, and cyclin D1 expression was obviously decreased by miR‐516a‐3p angomir compared with angomir control and PBS (Figure 7D). Additionally, the TUNEL assay results showed that the apoptosis rate of breast cancer tumour tissue increased after miR‐516a‐3p angomir treatment compared with angomir control and PBS (Figure 7E). IHC staining results showed that E‐cadherin protein was up‐regulated and Pygo2 protein and vimentin protein were down‐regulated in breast cancer tumour tissues treated with miR‐516a‐3p angomir (Figure 7F). These results suggest that miR‐516a‐3p inhibits breast tumour growth and EMT by blocking the Pygo2/Wnt signalling pathway in vivo.

## 4 DISCUSSION

Breast cancer is one of the most common malignancies, and metastasis is the major cause of death in breast cancer patients. Multiple reports have demonstrated the role of miRNAs in the breast cancer metastasis.^19^, ^30^, ^31^, ^32^ In this study, the results demonstrate that miR‐516a‐3p inhibits breast cancer cells growth, metastasis and EMT by suppressing the Pygo2/Wnt/β‐catenin signalling pathway (Figure 7G).

In this study, we found that miR‐516a‐3p expression was decreased and that of Pygo2 was up‐regulated in human breast cancer tissue and cells lines. To further explore the roles of miR‐516a‐3p and Pygo2 in breast cancer, we analysed the clinical significance of miR‐516a‐3p and Pygo2 expression in breast cancer patients. Our results showed that a low expression of miR‐516a‐3p or positive expression of Pygo2 was related to lymph node metastasis, increased tumour size, higher TNM stage and worse prognostic in breast cancer patients. These data indicated that both miR‐516a‐3p and Pygo2 have the potential to be used as prognostic biomarkers for breast cancer. Moreover, our study found that miR‐516a‐3p inhibited breast cancer cell proliferation, induced cell apoptosis in vitro and inhibited the progression of breast tumours in vivo. Previous studies have shown that miR‐516a‐3p expression is down‐regulated and has a similar tumour suppressor role in gastric cancer and ovarian cancer.^14^, ^15^ With respect to Pygo2, it has been confirmed as a tumour promoter in breast cancer,^22^ colorectal cancer,^33^ ovarian cancer,^34^ advanced prostate cancer^35^ and glioma.^36^ Zhou SY et al^37^ reported that Pygo2 was overexpressed in human lung cancer tissue samples and cell lines and that the knockdown of Pygo2 suppressed the growth of lung cancer in vitro and in vivo.

Bioinformatics analysis (http://mirtarbase.mbc.nctu.edu.tw/php/index.php and http://www.Targetscan.org) predicted that Pygo2 is a potential target gene of miR‐516a‐3p, which was confirmed by the results of a dual luciferase reporter assay. In this study, we found that cells, respectively, treated with miR‐516a‐3p mimic or inhibitor showed decreased or increased Pygo2 protein expression compared with control group. We confirmed that Pygo2 expression was attenuated in breast cancer tissues with high miR‐516a‐3p level compared with breast cancer tissues exhibiting low miR‐516a‐3p level through IHC staining analysis. These data further confirmed Pygo2 was a target of miR‐516a‐3p.

Epithelial‐mesenchymal transition is a well‐recognized process underlying breast cancer cell metastasis,^20^, ^38^, ^39^, ^40^ and E‐cadherin and vimentin are important EMT markers. E‐cadherin mediates cell adhesion and inhibits breast cancer metastasis, and the deletion of E‐cadherin is closely associated with advanced tumours. Vimentin is the main intermediate filament protein of mesenchymal cells. Up‐regulation of vimentin expression promotes breast cancer cell growth and metastasis. Interestingly, microRNAs showed important roles in EMT with miR‐200, miR‐10b, miR‐483‐5p, miR‐218 and miR‐127 have been proved to be crucial mediators of EMT.^20^, ^38^, ^41^, ^42^, ^43^ Our results demonstrated that miR‐516a‐3p inhibited breast cancer cell migration and invasion. miR‐516a‐3p overexpression increased E‐cadherin expression and decreased vimentin expression, and reduced levels of miR‐516a‐3p decreased E‐cadherin expression and increased vimentin expression relative to that in the control groups. The in vivo Western blot and IHC staining results were consistent with the in vitro. These results indicate that miR‐516a‐3p is a suppressor of metastasis and EMT in breast cancer.

Overexpression of Pygo2 enhanced hepatic carcinoma cell invasion and metastasis through reducing E‐cadherin expression. To explore the role of Pygo2 in the process of EMT regulated by miR‐516a‐3p, we increased Pygo2 expression through transfecting cells with pcDNA3.1‐Pygo2 plasmid. We found that increasing Pygo2 expression could reverse the effect of miR‐516a‐3p on the proliferation, migration, invasion and EMT of breast cancer cells. These results indicate that miR‐516a‐3p may inhibit cell proliferation, EMT and metastasis through blocking Pygo2.

It has been proved EMT is enhanced by the activation of the Wnt/β‐catenin signalling pathway^26^, ^44^ and abnormal expression of β‐catenin is closely related to the progression, metastasis and prognosis of breast cancer.^45^ Pygo2 combines with free β‐catenin to cause abnormal activation of downstream target genes, including c‐Myc and cyclin D1.^22^, ^25^ Downregulation of β‐catenin by siRNA inhibits cell proliferation and induces apoptosis.^46^ Studies have confirmed that c‐Myc and cyclin D1 were highly expressed in breast cancer.^47^, ^48^ Our data verified that miR‐516a‐3p suppressed the activity of the Wnt pathway by reducing β‐catenin, c‐Myc and cyclin D1 protein expression both in vitro and in vivo. We also found that miR‐516a‐3p induced cell apoptosis in vitro. These results demonstrate that miR‐516a‐3p inhibits breast cancer cell growth and metastasis through blocking the Wnt/β‐catenin pathway.

In this study, we explored the expression level, biological function and fundamental mechanisms of miR‐516a‐3p in breast cancer for the first time. We found that Pygo2 was a target of miR‐516a‐3p, and miR‐516a‐3p suppressed breast cancer cell growth and metastasis by inhibiting the Pygo2/Wnt/β‐catenin pathway, which were never reported. Either miR‐516a‐3p expression or Pygo2 expression was not related to molecular subtypes of breast cancer, but they were therapeutic targets and potential prognostic biomarkers of breast cancer.

In summary, we confirmed that the expression of miR‐516a‐3p was decreased and that of Pygo2 was increased in human breast cancer. The reduction of miR‐516a‐3p expression and the overexpression of Pygo2 were related to poor clinical features and a worse prognosis in breast cancer patients. Moreover, miR‐516a‐3p suppressed cell growth and EMT through blocking Pygo2 and inhibiting the Wnt pathway both in vitro and in vivo. These results provide insight into the molecular mechanism of miR‐516a‐3p in breast cancer cell growth and metastasis and demonstrate that miR‐516a‐3p may be a novel therapeutic target to treat breast cancer progression and metastasis.

## CONFLICT OF INTEREST

The authors confirm that there are no conflicts of interest.

## AUTHOR CONTRIBUTIONS

Yanyan Chi, Yan Sun and Qingxia Fan designed the research study; Yanyan Chi, Yanna Zhang, Zhengzheng Shan and Shaoxuan Wu performed the research; Feng Wang and Han Xu contributed essential reagents, tissues and animals; Yanyan Chi and Feng Wang analysed the data; Yanyan Chi wrote the paper; Feng Wang and Tengfei Zhang revised the manuscript. All authors approved the final version of the manuscript.
